# Preoperative (Neoadjuvant) Combined Chemoradiotherapy in the Management of Localized Soft Tissue Sarcoma: A Retrospective Study

**DOI:** 10.3390/cancers18081260

**Published:** 2026-04-16

**Authors:** Brittany L. Siontis, Georgios M. Stergiopoulos, Judith Jebastin Thangaiah, Thanh P. Ho, Safia K. Ahmed, Travis E. Grotz, Matthew T. Houdek, Andrew L. Folpe, Scott H. Okuno, Steven I. Robinson

**Affiliations:** 1 Department of Oncology, Mayo Clinic, 200 First Street SW, Rochester, MN 55905, USA; 2 Department of Molecular Medicine, Mayo Clinic, 200 First Street SW, Rochester, MN 55905, USA; 3 Department of Internal Medicine, Georgetown University, 3800 Reservoir Rd. NW, Washington, DC 20007, USA; 4 Department of Laboratory Medicine and Pathology, Mayo Clinic, 200 First Street SW, Rochester, MN 55905, USA; 5 Department of Radiation Oncology, Mayo Clinic Arizona, 5881 E. Mayo Blvd., Phoenix, AZ 85054, USA; 6 Department of Surgery, Mayo Clinic, 200 First Street SW, Rochester, MN 55905, USA; 7 Department of Orthopedic Surgery, Mayo Clinic, 200 First Street SW, Rochester, MN 55905, USA

**Keywords:** soft tissue sarcoma, sarcoma, combined chemoradiotherapy, neoadjuvant, chemotherapy, radiotherapy

## Abstract

**Background:** Localized soft tissue sarcoma (STS) is primarily treated with surgical resection with or without radiotherapy (RT), while the role of chemotherapy (CT) as a radiosensitizer remains unclear. We report our single-institution experience with combined chemoradiotherapy (CCRT) in treating localized STS. **Methods:** We conducted a retrospective analysis of patients with localized STS treated at Mayo Clinic with mitomycin, cisplatin, and doxorubicin (MitoAP) concurrently with RT between 1/1/85 and 12/12/19. **Results:** We identified 179 patients (median age 58 years; median tumor size 9.5 cm), with 83.8% of tumors located in the extremities or trunk. Among them, 77.1% received perioperative CT in addition to CCRT, with 95% of those treated in the neoadjuvant setting. Median RT dose was 50 Gray. The 5-year disease-specific survival (DSS) was 77.9% (95% confidence interval, CI: 70.8–83.4%). The addition of perioperative CT to CCRT was associated with improved DSS compared with CCRT alone (*p* = 0.01, Hazard Ratio, HR: 0.48, 95% CI: 0.27–0.85). Median post-CCRT tumor viability was 30% and did not differ by CT use (*p* = 0.39), but varied significantly by histology (*p* < 0.001). **Conclusions:** Our institutional protocol utilizing two cycles of MitoAP with RT was well tolerated. DSS in our cohort was similar to historical data using perioperative RT alone, suggesting no clear benefit from CCRT. However, the majority of patients in our cohort were classified as high risk, which may have attenuated a potential survival benefit in the absence of appropriate comparative controls. Furthermore, additional perioperative CT to CCRT was associated with improved DSS and differential histology-specific responses in tumor viability, suggesting that a more aggressive neoadjuvant and perioperative approach may be beneficial in selected patients.

## Simple Summary

This retrospective study reports outcomes of patients with localized soft tissue sarcoma (STS) treated with neoadjuvant combined chemoradiotherapy (CCRT) based on mitomycin, cisplatin, and doxorubicin (MitoAP), according to the Mayo Clinic institutional protocol. We identified 179 patients. Most patients received perioperative (adjuvant or neoadjuvant) chemotherapy in addition to CCRT. The addition of perioperative chemotherapy was associated with improved disease-specific survival, compared to patients receiving CCRT alone. The CCRT regimen was well tolerated. However, survival rates were comparable to historical outcomes using radiotherapy monotherapy.

## 1. Introduction

Soft tissue sarcoma (STS) comprises a heterogeneous group of rare malignancies arising from mesenchymal cells [1]. The Surveillance, Epidemiology, and End Results (SEER) database reports a 5-year overall survival (OS) rate of approximately 65% across all stages of STS, with localized disease showing significantly better outcomes, with a 5-year OS rate reaching 80% [2]. The primary treatment for patients with localized STS is complete surgical resection [3].

Neoadjuvant (NA) radiotherapy (RT) is the standard of care for patients in whom obtaining wide resection margins is not possible [3,4]. NA RT is preferred over adjuvant RT, due to potential benefits including a decreased radiation dose, shorter treatment time, and smaller field sizes [5,6]. Additionally, a retrospective multi-institutional analysis of 821 patients demonstrated superiority associated with NA compared to adjuvant RT [4].

The use of adjuvant chemotherapy (CT) for completely resected localized STS has long been an area of research, with initial data failing to demonstrate an additional survival benefit [7,8]. However, post hoc unplanned secondary analysis of EORTC-STBSG 62931 utilizing the Sarculator nomogram identified a survival benefit of adjuvant CT in high-risk patients [9]. NA CT for localized STS remains controversial but may be used to downsize locally advanced STS to enable complete surgical resection or to assess tumor aggressiveness and chemosensitivity in histologic subtypes with a high risk of distant metastasis, particularly before undertaking extensive, potentially morbid local surgery [10,11].

Various strategies have been explored to enhance the efficacy of NA RT for localized STS [11,12,13], none of which has yet provided sufficiently encouraging data on safety or efficacy to be adopted into standard practice [10]. Combined chemoradiotherapy (CCRT) is particularly attractive as CT not only has a direct anti-tumor effect but also acts as a sensitizer, enhancing STS responses to RT, potentially leading to a synergistic therapeutic benefit [12]. Multiple studies have been conducted over the years, but no consistent benefit from CCRT has been observed across STS in the NA setting [14,15,16,17,18,19,20,21]. Furthermore, the potential for wound healing complications in the context of CCRT must be considered [14,15]. Nonetheless, the National Comprehensive Cancer Network (NCCN) recognizes its utility without the guidelines specifying CT regimen(s) that should be considered [10].

Our institutional protocol for localized STS included two cycles of mitomycin, doxorubicin and cisplatin (MitoAP) to serve as a radiosensitizer during RT. In this study, we retrospectively identified patients with localized STS receiving NA CCRT with MitoAP treated at the Mayo Clinic Comprehensive Cancer Center (MCCC) and evaluated tumor response and clinical outcomes. To our knowledge, this represents the largest reported cohort of patients with localized STS treated with NA CCRT.

## 2. Materials and Methods

### 2.1. Study Population

We queried the MCCCC treatment records for patients who received NA CCRT with MitoAP for the treatment of localized STS from 1/1/85 to 12/12/19. Patients included in our cohort received therapy from 1998 to 2019. All patients underwent surgical resection following completion of the CCRT protocol. Patients considered for CCRT at our institution were typically those with aggressive histology, large (>5 cm) and high-grade tumors in deep anatomic locations.

### 2.2. Data Collection

Patient demographics, tumor location, histology, treatment details and outcomes were extracted from patient electronic medical records. Perioperative chemotherapy was defined as chemotherapy administered outside of the CCRT protocol. Neoadjuvant chemotherapy was administered prior to CCRT, while adjuvant therapy was administered postoperatively. Progressive disease (PD) was defined per the Response Evaluation Criteria in Solid Tumors (RECIST) criteria, as an increase of at least 20% in the sum of the diameters of target lesions, taking as reference the smallest sum on record (nadir), or the appearance of one or more new lesions.

Archival tumor specimens were reviewed by a bone and soft tissue pathologist who defined treatment response as the percent viable tumor, while blinded to the patients’ clinical outcome.

### 2.3. Statistical Analysis

Survival data were calculated using the Kaplan–Meier method. Statistical analysis was conducted with Stata (version 19, StataCorp LLC, College Station, TX, USA). Cox proportional hazards regression was used to estimate hazard ratios (HRs) with 95% confidence intervals (CIs). A *p*-value < 0.05 was considered statistically significant.

Differences in tumor viability in the surgical resection specimen for each STS histology were assessed using a Kruskal–Wallis statistical test. A *p*-value < 0.05 was considered statistically significant. Multivariate analysis utilizing a Cox proportional hazards model. Given the retrospective design, some data elements were not uniformly available for all patients. Missing data were handled using a complete-case analysis; patients with missing values for a given variable were excluded only from analysis involving that variable but retained for all others.

## 3. Results

### 3.1. Patient Demographics

We identified a total of 179 patients. The median age at diagnosis was 58 years (range: 18–83 years). A slight majority of the patients were male (n = 107, 59.8%). The median tumor size was 9.5 cm (range: 2.7 to 30 cm). Most of the tumors were located on the extremities or the trunk wall (n = 150, 83.8%), while fewer were retroperitoneal (n = 28, 15.6%), and only one was intrathoracic (n = 1, 0.6%). The most common histology was undifferentiated pleomorphic sarcoma/malignant fibrous histiocytoma (UPS/MFH, n = 61, 34.1%), followed by leiomyosarcoma, (LMS, n = 29, 16.2%), liposarcoma (LPS, n = 25, 14%; n = 10 pleomorphic LPS, n = 8 dedifferentiated LPS, n = 7 myxoid LPS, MLPS), synovial sarcoma (SS, n = 19, 10.6%), myxofibrosarcoma (n = 14, 7.8%), and malignant peripheral nerve sheath tumor (MPNST, n = 7, 3.9%). Other less common histologies (n = 24, 13.4%), such as high-grade sarcoma (n = 8), pleomorphic rhabdomyosarcoma (n = 4), angiosarcoma (n = 3), spindle cell sarcoma (n = 2), primitive neuroectodermal tumor (PNET, n = 2), fibrosarcoma (n = 1), extra-skeletal myxoid chondrosarcoma (n = 1), myofibroblastic tumor (n = 1), solitary fibrous tumor (n = 1), and malignant phyllodes tumor (n = 1) are presented collectively (Table 1).

### 3.2. Therapeutic Modalities

All patients included in our analysis received CCRT with MitoAP (n = 179, 100%) followed by surgical resection. The median NA RT dose was 50 Gray (Gy, range: 44–62.5 Gy). Most patients received the first dose of MitoAP on the same day as the first RT dose (median day difference: 0 days, 95% CI: 0 days). The median time from the last MitoAP infusion to surgery was 39 days (95% CI: 37–43 days), and the median time from the last RT dose to surgery was 33 days (95% CI: 30–35 days). Most patients achieved clear resection margins at surgery (n = 175, 97.8%), whereas the remaining patients had R1 resections (n = 4, 2.2%); no patients had an R2 resection.

In addition to CCRT, 138 patients (77.1%) received perioperative CT at the discretion of the treating oncologist, whereas 41 patients (22.9%) were treated only with CCRT. Baseline demographic and tumor characteristics of both groups are summarized in Table 2. The two cohorts were generally well-balanced with respect to age (median 58 years in both groups), tumor size (median 9.5 cm in both groups), sex distribution, and primary tumor location. The distribution of the most common histological subtypes was comparable between groups. Some differences were observed in the less prevalent subtypes: synovial sarcoma was more frequent in the CCRT+CT group compared with the CCRT-alone group (13% vs. 2.4%), whereas myxofibrosarcoma was more common in the CCRT-alone group (14.6% vs. 5.8%).

The majority of patients in the CCRT+CT group (n = 131, 95%) received CT in the NA setting prior to CCRT, with a median of two cycles (range: 1–6 cycles, Table 3). The most commonly used regimen was doxorubicin plus ifosfamide (AI) (n = 102, 77.9% of the NA CT cohort). Six patients received MitoAP prior to receiving the same regimen within the CCRT protocol. The best response to NA CT was stable disease (SD) per RECIST criteria (n = 101, 84.9% of the NA CT cohort). Fewer patients (n = 11, 6.1%) received CT in the adjuvant setting, with AI being the most common regimen (n = 9, 81.8% of the adjuvant CT cohort) and a median of four cycles (range: 2–4 cycles). The median time from last NA dose (start of the therapy cycle) to initiation of CCRT was 26 days (95% CI 25–28 days). The median cumulative anthracycline dose received in the MitoAP protocol was 60 mg/m^2^. Patients who received additional CT received a median of 120 mg/m^2^ of anthracycline for a cumulative dose of 180 mg/m^2^ for NA/A and CCRT.

### 3.3. Toxicity

One hundred sixty patients (89%) completed the planned course of two cycles of MitoAP concurrent with radiation. Of the 19 patients who completed only one cycle, 15 (79%) received neoadjuvant chemotherapy prior to commencement of the CCRT protocol. The most common reasons for early discontinuation included cytopenias (n = 7, 37%) and nausea (n = 2, 11%). The reason for discontinuation was not specified in six cases (32%). Of the four patients who received only CCRT, the reason for discontinuation was listed as not specified (n = 2), patient preference (n = 1), and pulmonary embolism (n = 1).

### 3.4. Disease-Specific Survival (DSS)

**Figure 1:**
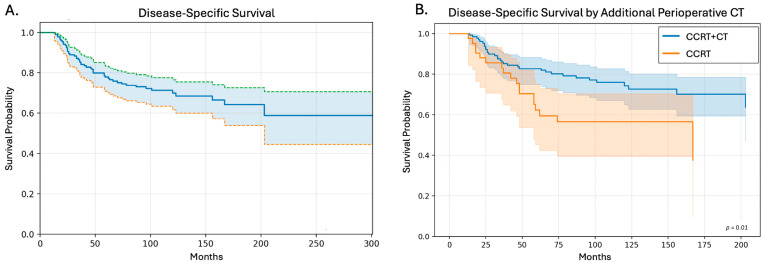
Disease-specific survival (DSS) Kaplan–Meier (KM) plots. (**A**) DSS KM plot for all patients with 95% CI (upper limit green, lower limit orange). (**B**) DSS for patients who received perioperative chemotherapy (CT) in addition to combined chemoradiation therapy (CCRT) vs. those who did not.

DSS outcomes are depicted in Figure 1. DSS for the overall cohort is presented in Figure 1A. Median DSS was not reached (NR). The 1-year, 3-year, 5-year, and 10-year DSS rates were 100%, 85.3% (95% CI 79.0–89.8), 77.9% (95% CI 70.8–83.4), and 69.9% (95% CI 61.7–76.7), respectively. Comparison of DSS for patients receiving CCRT+CT compared to CCRT was notable for prolonged DSS in the CCRT+CT group (*p* = 0.01, HR: 0.48, 95% CI: 0.27–0.85). The 5-year and 10-year DSS for the CCRT-alone group were 62.3% (95% CI: 45.4–75.4) and 56.5% (95% CI: 39.5–70.5) compared to the 5-year and 10-year DSS for the CCRT+CT which were 82.8% (95% CI: 75.1–88.3) and 74.3% (95% CI: 65–81.5), respectively. Those who only received CCRT had a median DSS of 167 months (95% CI: 58-NR) compared to CCRT+CT, where the median DSS was NR (95% CI: 203-NR). A multivariate analysis including tumor size, age at diagnosis, treatment era, tumor location, and histology showed no significant impact on DSS.

### 3.5. Relapse-Free Survival (RFS)

**Figure 2:**
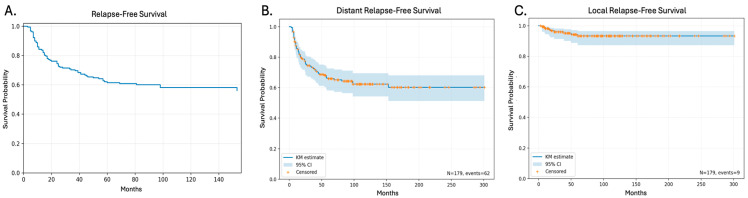
Relapse-free survival (RFS) Kaplan–Meier (KM) plots. (**A**) RFS for all patients. (**B**) Distant RFS (DRFS) for all patients. (**C**) Local RFS (LRFS) for all patients. Abbreviations: CI: confidence interval, KM: Kaplan–Meier.

Median RFS for all patients was NR, but 1-year RFS was 84.2% (95% CI: 77.9–88.8%), 5-year RFS was 70.2% (95% CI: 62.8–76.4%), and 10-year RFS was 58.1% (95% CI: 50.1–65.3%), as shown in Figure 2A. Most patients in our cohort had distant compared to localized relapse (62 vs. 9 events). The 5-year local RFS (LRFS) was 93.2% (95% CI: 87.3–96.5), and the 5-year distant RFS (DRFS) was 65.9% (95% CI: 58.1–72.6). The Kaplan–Meier plots for DRFS and LRFS are presented in Figure 2B and Figure 2C, respectively. Analysis of RFS by treatment period (1998–2005, 2006–2012, and 2013–2019) showed no significant difference in outcomes (*p* = 0.33). A multivariate analysis including tumor size, age at diagnosis, treatment era, tumor location, and histology identified tumor size as the only variable to impact RFS (*p* = 0.013).

### 3.6. Percent Tumor Viability by Tumor Histology and Treatment Effect

**Figure 3:**
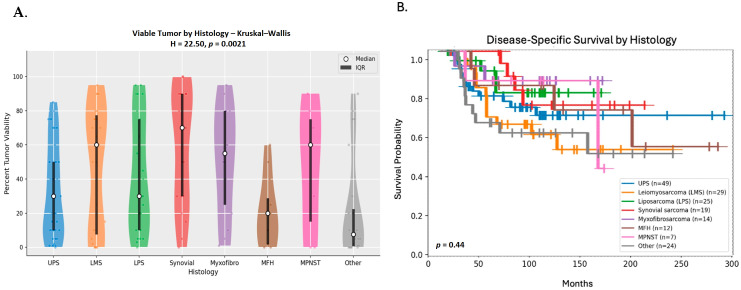
Tumor viability and disease-specific survival (DSS) by histology. (**A**) Percent viable tumor by histology. Median percent viable tumor was 20% (range: 0–85) for undifferentiated pleomorphic sarcoma/malignant fibrous histiocytoma (UPS/MFH), 60% (range: 0–95) for leiomyosarcoma (LMS), 30% (range: 0–95) for liposarcoma (LPS), 70% (range: 0–100) for synovial sarcoma (SS), 55% (range: 1–95) for myxofibrosarcoma, 60% (range: 0–90) for malignant peripheral nerve sheath tumor (MPNST) and 7.5% (range: 0–90, *p* < 0.002) for the “other” histologies. (**B**) Kaplan–Meier plot of DSS by histology. Abbreviations: LMS: leiomyosarcoma, LPS: liposarcoma, MPNST: malignant peripheral nerve sheath tumor, UPS/MFH: undifferentiated pleomorphic sarcoma/malignant fibrous histiocytoma.

The median percentage of viable tumor isolated from the surgical specimen post-CCRT for all patients was 30% (range: 0–100%). The median viable tumor for patients who received NA CT in addition to CCRT versus those who did not was 30% (range: 0–100%) and 45% (range: 0–95%), respectively (*p* = 0.39). The median viable tumor percentage varied significantly by histology (*p* < 0.002), as shown in Figure 3A. Median DSS varied as well as illustrated in Figure 3B, but the difference among the groups was not statistically significant (*p* = 0.44).

**Figure 4:**
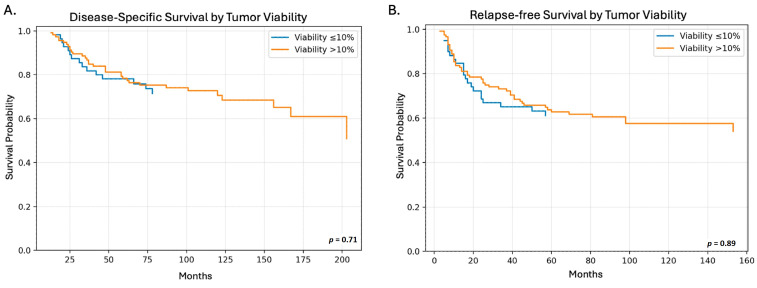
Kaplan–Meier plot of disease-specific survival (DSS) and relapse-free survival (RFS) stratified by >10% or ≤10% viable tumor by histology following combined chemoradiotherapy. (**A**) Kaplan–Meier plot for DSS by percent tumor viability. (**B**) Kaplan–Meier plot for RFS by percent tumor viability.

Additionally, we explored whether the percentage of viable tumor following CCRT could serve as a predictor of DSS or RFS following treatment. We used the 10% tumor viability cut-off, as this has historically been used for osteosarcoma [22,23]. No significant difference was observed in DSS (*p* = 0.71, HR: 1.12, 95% CI: 0.61–2.06, Figure 4A) or in RFS (*p* = 0.89, HR: 0.97, 95% CI: 0.58–1.6, Figure 4B).

## 4. Discussion

Our institutional CCRT protocol, combining two cycles of MitoAP radiosensitizing CT during NA RT, was found to be safe and well tolerated in patients with localized STS. Survival outcomes and local relapse rates observed in our cohort were comparable to those reported in historical series of patients treated with RT alone, although direct comparisons are limited by differences in patient populations and study design [4,24,25,26,27,28]. Notably, the 5-year DSS in our study was 77.9% (95% CI: 70.8–83.4%), while prior large retrospective cohorts of patients with localized STS receiving perioperative or NA RT reported rates of 73% (95% CI: 71–75%) and 79%, respectively [4,26]. Similarly, the 5-year DRFS in our study was not superior to historical outcomes reported for patients receiving neoadjuvant RT without MitoAP, reported as 71% in a recent meta-analysis [29].

Contrary to RT, which has an established role for localized STS, the use of adjuvant CT for localized STS remains controversial, as large randomized trials like the EORTC-STBSG 62931 have failed to demonstrate a survival benefit in the overall STS population [7,8]. However, post hoc secondary analysis of the same cohorts demonstrated a survival benefit of adjuvant CT in high-risk patients, as deemed by a predicted 10-year OS <60% by the Sarculator nomogram [9].

The observed trend of prolonged DSS associated with the use of perioperative CT (95% of which was NA) in our cohort may reflect a similar risk-dependent effect. When interpreted in the context of the above randomized clinical trial, our findings suggest that our cohort may overrepresent a higher-risk population compared to those included in prior studies. Notably, 54% of extremity or trunk STS patients in our cohort had a predicted 10-year OS < 60%, compared with 26% of patients in the EORTC-STBSG trial [9]. This difference in baseline risk may account for the improved DSS observed in our cohort with the addition of CT to CCRT, but also raises concerns regarding a potentially blunted survival benefit when comparing the overall cohort with historical data.

Histological analysis revealed significant variation in pathological response to CCRT across histologic subtypes. One of the most responsive histologies to NA CCRT was LPS, which not only showed lower post-resection percent tumor viability, but also appeared to have relatively longer DSS in comparison with other histologies like LMS. A similar trend in DSS was seen in SS, even though its median percent tumor viability was relatively high.

It has already been established that certain histologies, such as UPS/MFH, LPS (particularly MLPS) and SS, are more radio- and chemosensitive compared to other STSs such as LMS or MPNST [30,31,32]. Our findings are consistent with these observations.

NA histology-tailored CT has not been shown to meaningfully alter RFS in high-risk STS [33]; however, the established heterogeneous responsiveness of STS subtypes to different CT regimens represents a potential limitation in the interpretation of these data. For instance, in the largest retrospective review of patients with metastatic LMS treated with first-line CT, superior response rates were observed when doxorubicin was combined with dacarbazine compared to ifosfamide or doxorubicin alone [34]. In our cohort, none of the LMS patients received doxorubicin plus dacarbazine, which might account for the more resistant profile noted.

The potential of MitoAP CCRT in selected STS can be further highlighted by a recent case report published by our group focusing on a patient with a solitary cardiac metastasis from MLPS who received CT and CCRT based on MitoAP, resulting in stable disease for more than 10 years without surgical removal of the residual tumor [35]. Notably, MLPS is among the most radio- and chemosensitive STS, making it a particularly promising target for this approach [36,37]. Furthermore, our analysis hinted at an encouraging increase in DSS for SS following MitoAP-based CCRT. This is in accordance with the current literature considering SS to be a chemosensitive STS subtype, and builds on data indicating that SS sensitivity is not limited to alkylating agents, such as ifosfamide, but might extend to anthracyclines, like doxorubicin [30,31,32,38].

The rarity as well as varying response to CT and RT necessitate the identification of predictive biomarkers to guide treatment strategies for STS. One predictive marker for NA therapy response could be the percent tumor viability. Tumor viability has been consistently recognized for its prognostic and predictive value in bone sarcomas, and accumulating evidence suggests it could play a similar role in STS; however, there is no consensus on the exact viability percentage cut-off that is predictive for STS NA therapy response [11].

A meta-analysis evaluating 1663 patients from 21 studies undergoing different modalities of NA therapy (56% CCRT, 25% CT, 5% RT, 14% isolated limb perfusion, and 1% targeted therapy and RT) suggested that <90% necrosis in the tumor specimen post-NA treatment is associated with increased risk of recurrence (HR: 1.47, 95% CI: 1.06–2.04, *p* = 0.02) and death (HR: 1.86, 95% CI: 1.41–2.46, *p* < 0.001) [39]. However, certain studies have adopted a more stringent threshold, using pathologic complete response (0% tumor viability) as a predictor of survival benefit following NA therapy [40,41]. In our cohort, we applied a 10% tumor viability cut-off post-CCRT to explore its potential for predicting response, but this threshold did not prove effective for risk stratification. We explored alternative tumor viability thresholds for the potential to predict survival, none of which were significant.

Our study has several limitations, and its findings should therefore be interpreted with caution. First, its single-institution, retrospective design limits the ability to establish causal relationships and increases the potential for selection bias. Additionally, referral patterns at tertiary centers and administration of more aggressive regimens might have resulted in an overrepresentation of higher-risk patients, potentially skewing survival estimates toward poorer prognostic groups. Additionally, the rarity of STS often mandates their study collectively, even though each histology bears unique characteristics. Furthermore, the absence of a concurrent comparison group within our institution consisting of patients who did not receive CCRT limits the ability to accurately assess the relative effectiveness of CCRT in this study. In our cohort, most patients received perioperative CT, reducing the study’s internal validity for assessing the efficacy of preoperative CCRT with MitoAP. Additionally, some variables were incompletely captured in a minority of patients, reflecting the inherent constraints of retrospective data collection. Finally, we must acknowledge the impact that improvements in surgical and radiation techniques may have on outcomes, given that the included patients were treated over a 20-year period.

Future research aimed at improving RT outcomes in localized STS includes novel approaches, such as radiosensitizing nanoparticles or immunotherapy [24,42]. One especially promising nanoparticle, NBTXR3, has doubled the number of patients with locally advanced STS achieving pathological complete response following NA external beam RT [43,44]. A notable example of combining immunotherapy with RT is the randomized phase II clinical trial (NCT03092323) that evaluated NA pembrolizumab (anti–programmed cell death protein 1, PD-1) plus RT versus RT alone. The study demonstrated that the combination significantly improved RFS compared to RT alone in patients with UPS/MFH, dedifferentiated, or pleomorphic LPS [24]. The combination resulted in a 15% absolute increase in 2-year RFS (67% vs. 52%) with an HR of 0.61 (90% CI: 0.39–0.96, *p* = 0.04) [24]. Although grade ≥ 3 adverse events were more frequent with the addition of pembrolizumab [24], the RFS difference establishes this combination as a promising new treatment strategy for this patient population.

## 5. Conclusions

Our retrospective study evaluating the effect of NA CCRT based on two cycles of MitoAP in patients with localized STS indicated that it was well tolerated with minimal toxicity, as demonstrated by a high completion rate of 89%. A DSS benefit was observed in patients receiving perioperative CT (95% of whom were in the NA setting) in addition to CCRT. The Sarculator risk stratification indicated an increased representation of patients with poor predicted OS within our cohort, supporting the rationale for perioperative CT in high-risk patient populations. DSS in our cohort was similar to historical data using perioperative RT alone, suggesting no added benefit by CCRT. However, any potential incremental survival benefit may have been attenuated by the overrepresentation of high-risk patients in our study population. Overall, CCRT should not be considered standard of care for localized STS but may be considered in select clinical scenarios, such as in patients who are not surgical candidates or in cases where surgery would be associated with substantial morbidity and mortality (e.g., cardiac location). Importantly, our data demonstrated significant variability in treatment response between STS histologic subtypes, suggesting potential benefit for MitoAP CCRT in certain STS histologies such as LPS and SS, though multivariate analysis showed no impact of histology on survival. Future comparison of our cohort’s survival data with that from other institutions that did not implement a CCRT regimen, as well as conducting prospective studies using NA CCRT focusing on the most sensitive STS histologies, would be of value.

## Figures and Tables

**Table 1:** Patient demographics.

	N (%)
**Gender**	
**Male**	107 (60%)
**Female**	72 (40%)
**Age** (years) median (range)	58 (18–83)
**Tumor Size** (cm), median (range)	9.5 (2.7–30)
**Primary Tumor Location**	
Extremity/Trunk	150 (83.8%)
Retroperitoneum	28 (15.6%)
Intrathoracic	1 (0.6%)
**Histology**	
Undifferentiated pleomorphic sarcoma	61 (34.1%)
Leiomyosarcoma	29 (16.2%)
Liposarcoma	25 (14.0%)
Synovial sarcoma	19 (10.6%)
Myxofibrosarcoma	14 (7.8%)
Malignant peripheral nerve sheath tumor	7 (3.9%)
Other	24 (13.4%)

**Table 2:** Demographics of patients who received only combined chemoradiation therapy (CCRT) or CCRT + chemotherapy (neoadjuvant and/or adjuvant).

	CCRT + CT (n = 138)	CCRT with No CT (n = 41)
	N (%)	
**Gender**		
**Male**	84 (60.8%)	23 (56.1%)
**Female**	54 (39.1%)	18 (43.9%)
**Age** (years) median (range)	58 (18–76)	58 (32–83)
**Tumor Size** (cm), median (range)	9.5 (3–30)	9.5 (2.7–28)
**Primary Tumor Location**		
Extremity/Trunk	115 (83.3%)	35 (85.4%)
Retroperitoneum	22 (15.9%)	6 (14.6%)
Intrathoracic	1 (0.7%)	0 (0%)
**Histology**		
Undifferentiated pleomorphic sarcoma	49 (35.6%)	12 (29.3%)
Leiomyosarcoma	22 (15.9%)	7 (17.1%)
Liposarcoma	19 (13.7%)	6 (14.6%)
Synovial sarcoma	18 (13%)	1 (2.4%)
Myxofibrosarcoma	8 (5.8%)	6 (14.6%)
Malignant peripheral nerve sheath tumor	4 (2.9%)	3 (7.3%)
Other	18 (13%)	6 (14.6%)

Abbreviations: CCRT: combined chemoradiation therapy, CT: chemotherapy.

**Table 3:** Chemotherapy regimens used and response to therapy.

	N (%)
**Neoadjuvant Chemotherapy (n = 131)**	
AI	102 (77.9%)
IMAP	22 (16.8%)
MitoAP	6 (4.6%)
Doxorubicin	1 (0.8%)
**Median # of Neoadjuvant Cycles (range)**	2 (1–6)
**Median Cumulative Doxorubicin Dose (mg/m^2^, range)**	120 (60–360)
**Neoadjuvant Chemotherapy Best Response**	
Stable Disease	101 (84.9%)
Partial Response	11 (9.2%)
Progressive Disease	6 (5.0%)
Complete Response	1 (0.8%)
**Adjuvant Chemotherapy (** **n = 11)**	
AI	9 (81.8%)
IMAP	1 (9.1%)
IE	1 (9.1%)

Abbreviations: AI: doxorubicin/ifosfamide; IMAP: ifosfamide, mitomycin, doxorubicin, cisplatin; MitoAP: mitomycin, doxorubicin, cisplatin; IE: ifosfamide/etoposide.

## Data Availability

The data presented in this study are available on request from the corresponding author.

## Abbreviations

AI	Doxorubicin/Ifosfamide
ANOVA	Analysis of Variance
CCRT	Combined Chemoradiotherapy
CI	Confidence Interval
CT	Chemotherapy
DRFS	Distant Relapse-Free Survival
DSS	Disease-Specific Survival
HR	Hazard Ratio
Gy	Gray
IE	Ifosfamide/Etoposide
IMAP	Ifosfamide, Mitomycin, Doxorubicin, Cisplatin
KM	Kaplan–Meier
LMS	Leiomyosarcoma
LPS	Liposarcoma
LRFS	Local Relapse-Free Survival
MCCC	Mayo Clinic Comprehensive Cancer Center
MFH	Malignant Fibrous Histiocytoma
MitoAP	Mitomycin, Doxorubicin, Cisplatin
MLPS	Myxoid Liposarcoma
MPNST	Malignant Peripheral Nerve Sheath Tumor
NA	Neoadjuvant
NCCN	National Comprehensive Cancer Network
NR	Not Reached
OS	Overall Survival
PD	Progressive Disease
PD-1	Programmed Cell Death Protein 1
PNET	Primitive Neuroectodermal Tumor
RECIST	Response Evaluation Criteria in Solid Tumors
RFS	Relapse-Free Survival
RT	Radiotherapy
SD	Stable Disease
SEER	Surveillance, Epidemiology, and End Results
SS	Synovial Sarcoma
STS	Soft Tissue Sarcoma
UPS	Undifferentiated Pleomorphic Sarcoma
